# The Effects of COVID-19 Surges on Otolaryngology Consults

**DOI:** 10.7759/cureus.44794

**Published:** 2023-09-06

**Authors:** Evan B Hughes, Amanda E Gemmiti, Nadia Debick, Heidi Proper, Brian Nicholas, Amar Suryadevara

**Affiliations:** 1 Department of Otolaryngology-Head and Neck Surgery, State University of New York (SUNY) Upstate, Syracuse, USA

**Keywords:** public health, surge, consults, otolaryngology, covid-19

## Abstract

Objectives

We aimed to evaluate the effects COVID-19 surges had on an otolaryngology service’s consultation data.

Methods

After obtaining Upstate University Hospital institutional review board exemption to perform this research, a retrospective review analyzing otolaryngology consults at a single institution during COVID-19 surges in the years 2020 to 2021 was performed. The total consult volume and emergency department, inpatient, pediatric, adult, weekday, night, and weekend consults were assessed. Statistical analysis was used to compare these findings to the otolaryngology consult volumes and characteristics during the same time frames as the COVID-19 surges in the years 2014 to 2019.

Results

Based on bivariate analysis, an upward trend in otolaryngology consult volume was found over the study period. Although there was not a significant difference in consult volume during COVID-19 surges compared to historical data (p = 0.718, p = 0.695), both surge periods had significantly lower proportions of emergency department and pediatric consults (p < 0.001 for both).

Conclusion

Our study demonstrated that despite increasing cases of COVID-19 infection, otolaryngology consult volume remained high during surges. It was found that there has been an increase in otolaryngology consult volume at our academic center from the year 2014 to the present, a finding that was also seen in previous studies from our institution. Interestingly, consult parameters that changed when compared to the historical data included a decreased percentage of emergency department and pediatric consults during COVID-19 surges. The summation of these findings can be used to provide insight into how hospitals and otolaryngology services can prepare for the anticipated fluctuations in COVID-19 cases and associated hospitalizations.

## Introduction

The COVID-19 pandemic has changed many aspects of life, including the provision of medical care. Public transportation closures, stay-at-home orders, decreased healthcare service availability, and fear created barriers to care for those suffering from illnesses unrelated to COVID-19. Decreases in healthcare utilization were seen for both emergency and routine healthcare, including preventive care and care for chronic conditions [1]. Emergency general surgery in Milan, Italy, including injury-related emergency department admissions for surgery, decreased by 19% during the pandemic when compared to 2019 [2]. As COVID-19 has become an endemic disease, medical centers and hospital systems must prepare for seasonal waves in case numbers and the changes this may bring to healthcare utilization and delivery. The overall decrease in healthcare utilization has many potential effects, which may include decreased consult volume on a surgical subspecialty service. This study aims to determine the effects of COVID-19 on an otolaryngology consult service at an academic institution.

Prior to the pandemic, there had been a significant increase in the otolaryngology consult volume at our institution (the actual institution name was removed for blinding purposes). A 144% increase in consults was noted from March 1, 2014, to December 31, 2018. A total of 2,416 consults were seen in the final year of the study, compared to 990 consults seen during the first year [3]. This present study aims to evaluate the effects of surges in COVID-19 cases on otolaryngology consults at our institution (the actual institution name was removed for blinding purposes). There is conflicting data in the literature regarding the effects of COVID-19 on consult volume. At another academic institution, monthly consult numbers decreased by 21.5% after the start of the pandemic as compared to the prior six months. However, the demographics and chief complaints of patients seen were similar during both time periods [4]. Another institution recorded similar monthly consult numbers during the COVID-19 pandemic as the months prior, and the 10 most common chief complaints remained the same. However, these complaints became an increased proportion of overall consults. Additionally, the number of ICU consults increased and the number of ER and floor consults decreased. Airway consults also increased and rhinology, otology, and head and neck consults decreased [5]. Given the institutional differences and lack of robust research on COVID-19 effects on otolaryngology consults, a retrospective review was conducted to evaluate how COVID-19 has affected otolaryngology consults at our institution (the actual institution name was removed for blinding purposes). This review analyzes consult characteristics and consult volume during surge periods in New York state (March 1, 2020, to May 15, 2020, and November 12, 2020, to January 27, 2021) and compares the data to consults seen during the same time frames in the years 2014-2019. Data from 2014 to 2019 was used for comparison as the number of and reason for consults was most accurate after the implementation of the electronic medical record at our institution.

This research was presented at the Pan American Otolaryngology: Head and Neck Surgery Conference on June 26, 2022.

## Materials and methods

Study design

Data regarding ENT consult volume at Upstate Medical University from 2014 to 2021 was extracted from electronic medical records for retrospective review. Historical data from previous years was compared with data from COVID-19 surge periods, from March 2020 to May 2020 and from November 2020 to January 2021, to assess the influence of the COVID-19 pandemic on ENT consult volume. Of note, only physical consults were included for analysis.

Variables and data analysis

The primary dependent variable for this study was the mean number of ENT consults per week. The primary independent variable was the presence or absence of a COVID-19 surge period, defined as March 2020 to May 2020 and November 2020 to January 2021. Several key covariates were included for analysis to assess for differences between historical data and surge periods. Data was collected regarding the location of the consult, either in the emergency department or another setting, including inpatient medicine and clinic sites. Age was included as either pediatric or adult, with patients less than 18 years defined as pediatric. Race was included for analysis, and categories included American Indian, Asian, Black, Hispanic, other, unknown, and White. Time of consult was included as two categories: 7:00 to 17:00 and 17:01 to 6:59. The day of consult was noted and categorized as weekday (Monday to Friday) or weekend (Saturday and Sunday). Sex was included as male or female. Age in years was included as a continuous variable, with individuals older than 89 years top-coded to protect patient identity. Bivariate testing was conducted to assess for differences in consult volume and covariates between historical data and surge periods and included independent samples T-tests and chi-square tests.

## Results

Sample characteristics

**Figure 1:**
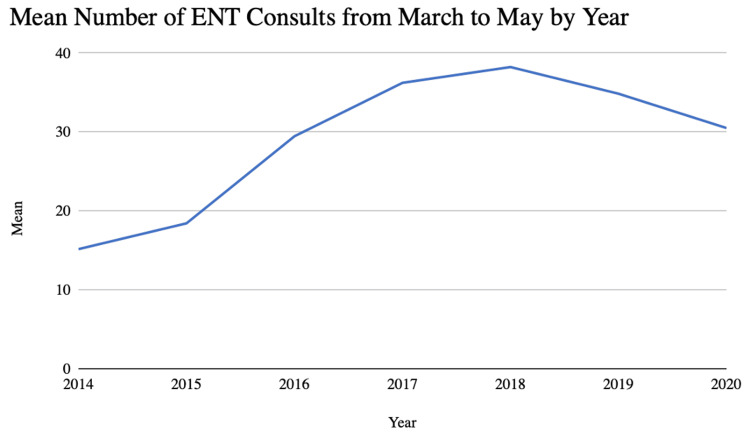
Mean Number of ENT Consults Per Week From March to May ENT: ear, nose, and throat

**Figure 2:**
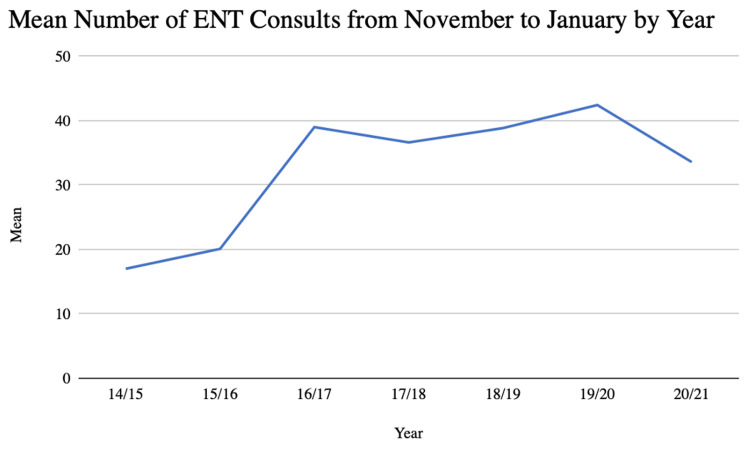
Mean Number of ENT Consults Per Week From November to January ENT: ear, nose, and throat

Overall, the mean number of ENT consults per week during this study was 30.74 (Table 1). The mean value of ENT consults per week during the March to May and November to January surge periods were 30.5 and 33.6, respectively (Figures 1, 2). The majority (84.8%) of patients seen by the ENT consult service were seen through the emergency department. Adult-aged patients contributed to 74.6% of the sample, with pediatric-aged patients contributing the remaining 25.4% of the sample. The most commonly represented race was White, with 4,156 of the patients or 75.7% of the sample identifying as White. The second most commonly represented race was Black, with 815 patients or 14.9% of the population identifying as Black. Patients who identified as American Indian, Asian, Hispanic, or other or whose race was unknown contributed to the final 9.4% of the sample. Of the patients seen through the ENT consult service, 72.5% were seen on a weekday, and 50.1% were seen between the hours of 7:00 and 17:00. Males comprised 59.3% of the sample. The mean age at the time of consult within the sample was 39.1 years.

**Table 1:** Sample Characteristics of Patients Seen Through the ENT Consult Service From 2014 to 2021 ENT: ear, nose, and throat, SD: standard deviation

	Number (%)
Emergency Department	
No	837 (15.2)
Yes	4,657 (84.8)
Age Group	
Pediatric	1,393 (25.4)
Adult	4,098 (74.6)
Race	
American Indian	51 (0.9)
Asian	32 (0.6)
Black	815 (14.9)
Hispanic	22 (0.4)
Other	336 (6.1)
Unknown	75 (1.4)
White	4,156 (75.7)
Weekday	
No	1,513 (27.5)
Yes	3,982 (72.5)
Time	
7:00 to 17:00	2,753 (50.1)
17:01 to 6:59	2,742 (49.9)
Sex	
Male	3,257 (59.3)
Female	2,238 (40.7)
	Mean (SD)
Age in Years	39.1 (25.6)

Statistical analysis

When compared to “March-May 2014,” ENT consult volumes per week were significantly higher during the “March-May” period during the surge (mean difference = -15.35, 95% confidence interval (CI) (-23.20, -7.50), p < 0.001) (Table 2). When compared to “March-May 2015,” ENT consult volumes per week were significantly higher during the “March-May” period during the surge (mean difference = -12.08, 95% CI (-20.69, -3.48), p = 0.008). When compared to “November 2014-January 2015,” ENT consult volumes per week were significantly higher during the “November-January” surge period (mean difference = -16.57, 95% CI (-24.04, -9.11), p < 0.001) (Table 3). When compared to “November 2015-January 2016,” ENT consult volumes per week were significantly higher during the “November-January surge period” (mean difference = - 13.48, 95% CI (-22.60, -4.36), p = 0.006). During the “March-May surge period,” consults were significantly less likely to be seen through the emergency department when compared to historical data (χ2 (1) = 41.5, p < 0.001) (Table 4). Patients seen as consults during the “March-May surge period” were significantly more likely to be adult-aged when compared to historical data (χ2 (1) = 12, p < 0.001). There was an association between race and consult period (χ2 (6) = 108.0, p < 0.001). The data suggests that patients through the ENT consult seen during the “March-May surge period” were more likely to identify as White. Additionally, patients seen as consults during the “March-May surge period” were on average significantly older than their counterparts seen in historical years (t (2,570) = -4.37, p < 0.001). During the “November-January surge period,” consults were significantly less likely to be seen through the emergency department when compared to historical data (χ2 (1) = 45.1, p < 0.001) (Table 5). Patients seen as consults during the “November-January surge period” were significantly more likely to be adult-aged when compared to historical data (χ2 (1) = 7.3, p = 0.007). Lastly, patients seen as consults during the “November-January surge period” were on average significantly older than their counterparts seen in historical years (t (2,910) = -2.16, p = 0.029). The percentage of COVID-19-positive ENT consults was significantly higher during the “November-January surge” when compared to the “March-May surge,” at 5.3% and 2.5%, respectively (p = 0.046).

**Table 2:** March to April Mean Number of Consults per Week (2014-2019) Compared to Surge Period (2020) df: degrees of freedom, CI: confidence interval

Year	t value	df	Mean Difference	95% CI (Lower)	95% CI (Upper)	p-value
2014	-4.04	23	-15.35	-23.20	-7.50	<0.001
2015	-2.911	22	-12.08	-20.69	-3.48	0.008
2016	-0.201	23	-1.04	-11.71	9.64	0.842
2017	1.046	23	5.73	-5.60	17.06	0.306
2018	1.519	23	7.73	-2.80	18.26	0.142
2019	0.825	23	4.35	-6.55	15.23	0.418

**Table 3:** November to January Mean Number of Consults per Week (2014-2020) Compared to Surge (2020-2021) df: degrees of freedom, CI: confidence interval

Year	t value	df	Mean Difference	95% CI (Lower)	95% CI (Upper)	p-value
2014/2015	-4.61	22	-16.57	-24.04	-9.11	<0.001
2015/2016	-3.057	23	-13.481	-22.60	-4.36	0.006
2016/2017	1.271	22	5.43	-3.43	14.28	0.217
2017/2018	0.601	25	3.04	-7.39	13.48	0.553
2018/2019	0.986	25	5.28	-5.75	16.30	0.334
2019/2020	1.650	26	8.86	-2.188	19.89	0.111

**Table 4:** Comparison of Consult Characteristics Between Historical March-May (2014-2019) Periods and Surge March-May (2020) Period df: degrees of freedom

	Historical March-May (Number (%))	Surge March-May (Number (%))	χ^2^	df	p-value
Emergency Department			41.5	1	<0.001
No	263 (11.8)	87 (24.4)			
Yes	1,959 (88.2)	269 (75.6)			
Age Group			12.0	1	<0.001
Pediatric	604 (27.2)	65 (18.5)			
Adult	1,618 (72.8)	287 (81.5)			
Race			108.0	6	<0.001
American Indian	21 (0.9)	2 (0.6)			
Asian	14 (0.6)	5 (1.4)			
Black	343 (15.4)	44 (12.4)			
Hispanic	1 (0)	15 (4.2)			
Other	149 (6.7)	8 (2.2)			
Unknown	39 (1.8)	0 (0)			
White	1,655 (74.5)	282 (79.2)			
Weekday			0.057	1	0.811
No	619 (27.9)	97 (27.2)			
Yes	1,603 (72.1)	259 (72.8)			
Time			0	1	0.986
7:00 to 17:00	1,010 (45.5)	162 (45.5)			
17:01 to 6:59	1,212 (54.5)	194 (54.5)			
Sex			0.187	1	0.665
Male	1,319 (59.4)	207 (58.1)			
Female	903 (40.6)	149 (41.9)			

**Table 5:** Comparison of Consult Characteristics Between Historical November-January (2014-2020) Periods and Surge (November 2020-January 2021) Period df: degrees of freedom

	Historical November-January (Number (%))	Surge November-January (Number (%))	χ^2^	df	p-value
Emergency Department			45.1	1	<0.001
No	363 (14.7)	124 (27.6)			
Yes	2,103 (85.3)	326 (72.4)			
Age Group			7.3	1	0.007
Pediatric	635 (25.7)	89 (19.8)			
Adult	1,832 (74.3)	361 (80.2)			
Race			2.4	6	0.882
American Indian	23 (0.9)	5 (1.1)			
Asian	12 (0.5)	1 (0.2)			
Black	363 (14.8)	65 (14.5)			
Hispanic	6 (0.2)	0 (0)			
Other	148 (6.00)	31 (6.9)			
Unknown	30 (1.2)	6 (1.3)			
White	1,878 (76.3)	341 (75.9)			
Weekday			0.01	1	0.913
No	675 (27.4)	122 (27.1)			
Yes	1,792 (72.6)	328 (72.9)			
Time			2.7	1	0.098
7:00 to 17:00	1,321 (53.5)	260 (57.8)			
17:01 to 6:59	1,146 (46.5)	190 (42.2)			
Sex			3.5	1	0.061
Male	1,446 (58.6)	285 (63.3)			
Female	1,021 (41.4)	165 (36.7)			

Overall, patients seen by the ENT consult service during COVID-19 surge periods were significantly less likely to be seen through the emergency department, significantly more likely to be adult-aged, and on average significantly older than their counterparts seen during the same time frame in previous years. These findings were observed both during the “March-May” and “November-January” surge periods.

## Discussion

The COVID-19 pandemic has had unprecedented effects on societies and healthcare systems across the world. Hospitals face the ongoing challenges associated with providing the necessary care for patients while balancing the risk of exposure with the use of valuable healthcare resources. The present study aimed to evaluate the impact of the pandemic on an academic otolaryngology service during time periods of increased COVID-19 infection rates but subsequently decreased ED visits, hospital admissions, and otolaryngology surgical volume [6-8].

Our academic hospital is a level 1 trauma center in upstate New York with a catchment area that includes 14 counties and 1.7 million people [9]. Previously reported studies out of our institution demonstrated an increased otolaryngology consult volume in the years just prior to the current study. During the COVID-19 pandemic, another study found that the incidence of facial trauma presenting to our hospital decreased during the COVID-19 statewide lockdown orders but remained unchanged after these orders were lifted, but COVID-19 rates remained high in the region [10]. Decreased trauma and facial trauma rates during COVID-19 peaks are well documented in the literature and have been considered to be effects of social distancing and stay-at-home directives [11,12].

The present study aimed to establish trends in overall otolaryngology consult volume at our academic medical center during periods of COVID-19 outbreaks in our community. Our results showed that despite rapidly increasing cases of COVID-19 infection, as well as statewide lockdowns and county restrictions, otolaryngology consult volume remained high during COVID-19 surges, with an average of over 40 consults per week. When these findings were compared to our historical data, the consult volumes during COVID-19 surges were noted to have increased from previous years. Although this may initially seem surprising, it is consistent with the trend of increased consult volumes since 2014 seen at our institution in previous studies [3].

Notably, the results also illustrate that there was an increased percentage of consults for COVID-19-positive patients during surge #2 compared to surge #1. This is potentially secondary to an increased understanding of the virus, especially as it pertains to the role of otolaryngologists, as well as readily available PPE. Early in the pandemic, tracheostomies were not recommended until several weeks after intubation and preferably with a negative COVID-19 test [13,14]. These recommendations were in place to reduce the risk of disease transmission to healthcare providers but also the lack of data regarding the survival benefit of tracheostomy placement in severely ill COVID-19 patients [15]. However, as more data was collected, studies found that early tracheostomy placement was associated with shorter hospital lengths of stay and was associated with low rates of transmission to providers [16]. Nonetheless, a relatively small percentage of the overall consults in our cohort were for COVID-19-positive patients despite the high rates of COVID-19 hospitalizations.

When comparing the type of consults placed during COVID-19 surges with the historical data, an increased percentage was found to be inpatient consults when compared to consults placed from the ED. This finding corresponds with the decreased rate of ED visits during COVID peaks, which has been suspected to be a result of the culmination of public concern about exposure to COVID-19 infection, the implementation of strict social distancing guidelines, and the subsequently reduced rate of trauma [5,10-12]. No statistically significant differences were found in regard to the timing or weekday consultation rates.

A higher proportion of otolaryngology consultations for adult patients when compared to pediatric patients during both surges was found when compared to historical data. Additionally, the average age of consult patients was seen to be higher during surges. These findings coincide with Centers for Disease Control and Prevention (CDC) reports of the largest declines in ED visits during the early pandemic period being in patients ≤14 years old [5]. No statistically significant differences were found regarding other consult patient characteristics such as race and gender.

There are several limitations to this study. First, due to its retrospective nature, data was collected by reviewing electronic medical records and is therefore susceptible to inaccuracies and reporting errors. Additionally, this study was limited to a single otolaryngology department’s experience at an academic institution under the guidance of New York state-specific directives during COVID-19 surges. These study factors, along with a relatively small sample size, may make the generalization and reproducibility of the identified consult volume trends difficult.

## Conclusions

Amidst strict social distancing guidelines and lockdown orders during COVID-19 surges, otolaryngology consult volume remained high. Certain characteristics of otolaryngology consults were found to vary when compared to pre-pandemic times and as the pandemic progressed. The summation of these findings can be used to provide insight into how hospitals and otolaryngology services can prepare for anticipated fluctuations in cases and associated hospitalizations as COVID-19 is predicted to become an endemic disease.
